# Post-mortem Findings of Inflammatory Cells and the Association of 4-Hydroxynonenal with Systemic Vascular and Oxidative Stress in Lethal COVID-19 †

**DOI:** 10.3390/cells11030444

**Published:** 2022-01-27

**Authors:** Neven Zarkovic, Antonia Jakovcevic, Ana Mataic, Morana Jaganjac, Tea Vukovic, Georg Waeg, Kamelija Zarkovic

**Affiliations:** 1 Laboratory for Oxidative Stress (LabOS), Ruder Boskovic Institute, HR-10000 Zagreb, Croatia; Morana.Jaganjac@irb.hr (M.J.); Tea.Vukovic@irb.hr (T.V.); 2 Clinical Hospital Centre Zagreb, Division of Pathology, HR-10000 Zagreb, Croatia; antonia.jakovcevic@gmail.com (A.J.); ana.mataic@gmail.com (A.M.); kamelijazarkovic@gmail.com (K.Z.); 3 Institute of Molecular Biosciences, Karl Franzens University, A-8010 Graz, Austria; georg.waeg@uni-graz.at; 4 Division of Pathology, University of Zagreb School of Medicine, HR-10000 Zagreb, Croatia

**Keywords:** SARS-CoV-2 virus, reactive oxygen species (ROS), oxidative stress, vascular stress, lipid peroxidation, 4-hydroxynonenal (4-HNE), superoxide dismutase (SOD), granulocytes, sepsis

## Abstract

A recent comparison of clinical and inflammatory parameters, together with biomarkers of oxidative stress, in patients who died from aggressive COVID-19 and survivors suggested that the lipid peroxidation product 4-hydroxynonenal (4-HNE) might be detrimental in lethal SARS-CoV-2 infection. The current study further explores the involvement of inflammatory cells, systemic vascular stress, and 4-HNE in lethal COVID-19 using specific immunohistochemical analyses of the inflammatory cells within the vital organs obtained by autopsy of nine patients who died from aggressive SAR-CoV-2 infection. Besides 4-HNE, myeloperoxidase (MPO) and mitochondrial superoxide dismutase (SOD2) were analyzed alongside standard leukocyte biomarkers (CDs). All the immunohistochemical slides were simultaneously prepared for each analyzed biomarker. The results revealed abundant 4-HNE in the vital organs, but the primary origin of 4-HNE was sepsis-like vascular stress, not an oxidative burst of the inflammatory cells. In particular, inflammatory cells were often negative for 4-HNE, while blood vessels were always very strongly immunopositive, as was edematous tissue even in the absence of inflammatory cells. The most affected organs were the lungs with diffuse alveolar damage and the brain with edema and reactive astrocytes, whereas despite acute tubular necrosis, 4-HNE was not abundant in the kidneys, which had prominent SOD2. Although SOD2 in most cases gave strong immunohistochemical positivity similar to 4-HNE, unlike 4-HNE, it was always limited to the cells, as was MPO. Due to their differential expressions in blood vessels, inflammatory cells, and the kidneys, we think that SOD2 could, together with 4-HNE, be a potential link between a malfunctioning immune system, oxidative stress, and vascular stress in lethal COVID-19.

## 1. Introduction

SARS-CoV-2 infection is associated with inflammation, redox imbalance, and elevated cytokine production [1,2]. In response to SARS-CoV-2 infection, cellular components of innate immunity are activated, contributing to increased reactive oxygen species (ROS) and further impairment of the redox balance [3]. COVID-19 patients with the biomedical pre-conditions where the redox balance is already altered are prone to developing severe symptoms. ROS take part in a number of redox signaling processes, can induce oxidation of macromolecules [4], and result in organelle dysfunction. Thus, tight control of the redox balance is maintained by antioxidant defense systems [5]. Myeloperoxidase (MPO), a mediator of inflammation and oxidative stress, was found to be several-fold upregulated in the lungs and whole blood of COVID-19 patients [6]. Moreover, following SARS-CoV-2 infection, both mitochondrial superoxide dismutase (SOD2) and heme oxygenase-1 (HO-1) were found to be altered, but their involvement in the pathogenesis of COVID-19 is not clear. SOD2 is among the key endogenous antioxidants, while HO-1 both attenuates oxidative stress and inhibits the inflammatory response. The induction of HO-1 was shown to suppress SARS-CoV-2 in vitro [7], but it is also significantly elevated in patients with severe COVID-19 (oxygen saturation level ≤95%) [8]. Strong upregulation of HO-1 suggests the involvement of both canonical and non-canonical ferroptosis pathway-mediated cell death [9]. Furthermore, SOD2 is decreased in low-density neutrophils [6], which correlates with hypercoagulation and COVID-19 severity [10].

SARS-CoV-2-induced mitochondrial dysfunction and excessive ROS may induce the activation of nucleotide-binding oligomerization domain-like receptor P3 (NLRP3) inflammasome and stimulate the release of proinflammatory cytokines [11,12]. Indeed SARS-CoV-2 infection promotes the activation of the NLRP3 inflammasome and is associated with COVID-19 severity [13]. In particular, SARS-CoV-2 nucleocapsid protein (N) is directly involved in the activation of the NLRP3 inflammasome triggering a cytokine “storm” [14], which may end with multi-organ dysfunction, severe acute respiratory syndrome (SARS), and death [15].

However, our preliminary findings obtained by a comparison of clinical and inflammatory parameters, together with biomarkers of oxidative stress, in patients who died due to aggressive COVID-19 and survivors suggested that lipid peroxidation might be detrimental in lethal SARS-CoV-2 infection [16]. Polyunsaturated fatty acids are particularly sensitive to ROS, triggering a self-catalyzed chain reaction of lipid peroxidation yielding reactive aldehydes, among which the most bioactive is 4-hydroxynonenal (4-HNE) [17]. 4-HNE is a well-known modulator of cellular functions [18] and was found to induce NLRP3, resulting in inflammasome activation [19], while it is also acknowledged as a reliable biomarker of ferroptosis, occurring also in lethal COVID-19 [20]. Through the activation of the extracellular signal-regulated kinases (ERK) pathway, 4-HNE upregulates HO-1 [21], while its effect on SOD2 depends on the duration of exposure [22]. On the other hand, 4-HNE has high affinity for binding to proteins, consequently changing their structure and function, but still maintaining its important role of the “second messenger of ROS”, which is relevant for various acute and chronic diseases and their respective therapies [18,23]. Our preliminary findings revealed the association of 4-HNE with lethal outcomes in COVID-19 patients, reflecting altered redox homeostasis and sepsis-like systemic vascular and oxidative stress mediated by the 4-HNE protein adducts [16], not being associated with the cytokine storm or even inflammation itself. Therefore, the roles of 4-HNE in the pathology of COVID-19 remain to be further explored.

This study aimed to provide a better understanding regarding the involvement of inflammation, systemic vascular stress, and 4-HNE in lethal COVID-19 as revealed by the immunohistochemical analyses of the vital organs obtained by autopsy of patients who died from aggressive SAR-CoV-2 infection.

## 2. Materials and Methods

### 2.1. Patients

The cases included in this study were nine COVID-19 patients, extracted from the case records of patients who died and underwent post-mortem examination according to the requests of their families. There were seven male patients and one female, with an average age of 62.9 ± 7.7 years, and one child of 11 years. All the patients were hospitalized at the intensive care unit of the Clinical Hospital Centre (KBC) Zagreb or Clinic for Infectious Diseases in Zagreb. All of them required oxygen supply, while two were supported by extracorporeal membrane oxygenation (ECMO). Swabs were regularly tested, showing that all patients were positive for SARS-CoV-2 before death. The study was approved by the Ethical Committee of the KBC Zagreb (approval code: 02/21 AG).

Because SARS-CoV-2 is classified as a biosafety level 3 (BSL3) risk, the autopsies were performed in a separate autopsy room, resembling the safe management strategies described by Pomara et al. [24], following the recommendations provided by the Center for Disease Control and Prevention (CDC) [25] and the World Health Organization (WHO) [26]. Personal protective equipment, including filtering face piece 2 (FFP2) masks, protective suits, and cut-resistant gloves were used, while a handsaw combined with chain mail gloves was used to saw the skull. The autopsies were carried out by pathologists experienced in BSL3 autopsies, who conducted postmortem evaluations by an in situ evaluation of the body cavities and organs, but a post-mortem swab of the lower airways was not taken because the patients has tested daily and were SARS-CoV-2 positive until death. All autopsies were performed between 17 and 28 h after death, assuring the accuracy of the evaluation and a sufficiently safe autopsy procedure [27,28].

### 2.2. Immunohistochemistry

Immunohistochemical analyses were performed for the tissue specimens obtained by autopsy performed within hours after the death. The specimens were fixed in 10% buffered formalin immediately after resection, dehydrated in ethanol, and embedded in paraffin. Paraffin blocks were cut into five-micron-thin slices and examined by section staining with hematoxylin and eosin, followed by immunohistochemistry. For the immunohistochemical analysis, the paraffin sections were deparaffinized, rehydrated, and subsequently quenched for endogenous peroxidase activity with a 3% hydrogen peroxide solution in phosphate buffered saline (PBS) at room temperature for 15 min. The sections were then incubated for two hours at room temperature with murine monoclonal antibodies against 4-HNE protein adducts as follows.

Immunohistochemical staining for the 4-HNE-modified proteins was performed using genuine monoclonal antibodies specific for the detection of the 4-HNE-histidine adducts, which were obtained from the culture medium of the clone derived from a fusion of Sp2-Ag8 myeloma cells with the B-cells of a BALBc mouse immunized by 4-HNE-modified keyhole limpet hemocyanin [29].

Dilutions of the antibody solution (1:10) and the appropriate reagents from the EnVision detection kit (K 8000, Agilent, Santa Clara, CA, USA) were used as previously described [30]. Antigens were localized using the avidin-biotin method with 3, 3,-diaminobenzidine (DAB) as a chromogen, and counterstained with hematoxylin (Kemika, Zagreb, Croatia). The DAB was used as chromophore, giving a brown-colored reaction in the case of a positive immunohistochemical reaction for the 4-HNE-histidine, with blue-colored contrast staining of hematoxylin. The immunohistochemical evaluation of the intensity and distribution of 4-HNE in the specimens of vital organs (lungs, heart, brain, liver, and kidneys) was classified as negative (in case of the absence of immunopositivity), low, moderate, and strong by two pathologists with expertise in the field who analyzed each specimen independently as described previously [31].

Serial cuts were also prepared for the hematoxylin-eosin stained (HE) slides and for the immunohistochemical evaluation of several other biomarkers including SOD2 (SIGMA, St. Luis, USA), cytokeratin (AE1AE3), and MPO (DAKO, Agilent, Santa Clara, CA, USA), which were all used alongside the immunostainings for the standard leukocyte biomarkers CD3 for T lymphocytes, CD20 for B-lymphocytes, CD138 for plasma cells, and CD68 for macrophages, according to the same manufacturer’s instructions (DAKO, Agilent, Santa Clara, CA, USA), and also following the analytical procedure as described for the 4-HNE-immunohistochemsitry.

All the tissues of each patient were processed simultaneously from autopsy to paraffin blocks. The immunohistochemical slides prepared from paraffin blocks were processed simultaneously for all organs and for all patients, but separately for each biomarker. Negative control slides were prepared without primary antibodies against 4-HNE and SOD2 (the negative controls are presented in the Supplementary Material), while the routine clinical biomarkers of leukocytes were analyzed in comparison to the human appendix control specimen.

All the immunohistochemical slides were simultaneously prepared for each particular biomarker analyzed, while the analyses of the obtained slides were conducted independently by three experienced pathologists.

## 3. Results

The major autopsy findings of the deceased COVID-19 patients are summarized in Table 1. While two patients died without any comorbidity combined with SARS-CoV-2 infection, one boy developed myocarditis combined with multiple inflammatory syndrome (MIS) and multiple organ failure (MOF) alongside COVID-19. The other patients were often obese and suffered mostly from high blood pressure and diabetes mellitus type 2. Aggressive SARS-CoV-2 infection manifested mostly as pneumonia, sepsis, and acute respiratory distress syndrome (ARDS).

**Figure 1:**
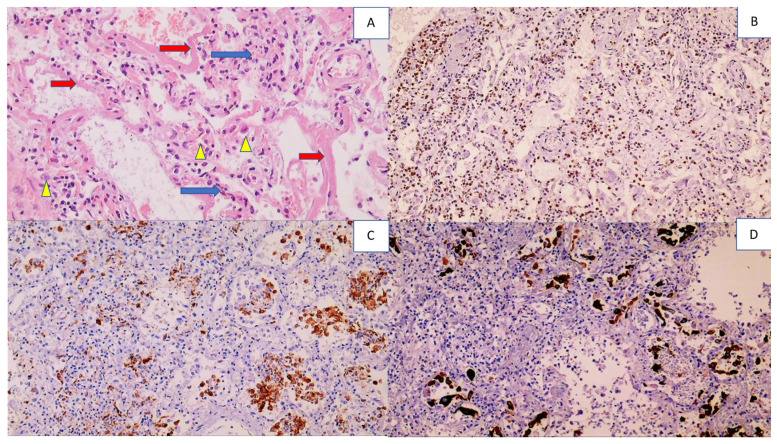
Histology and immunohistochemistry of the lungs with diffuse alveolar damage (DAD). Photo (**A**) shows lymphocyte-infiltrated alveolar septa (blue arrows), hyaline membranes lining the alveolar septa (red arrows), and alveolar macrophages (yellow arrowheads), (HE, (hematoxylin-eosin staining) 200×). Photo (**B**) shows infiltration of immunohistochemically CD3-positive T lymphocytes in the alveoli (DAB (3, 3,-diaminobenzidine) positivity presented in brown, with blue contrast staining with hematoxylin 100×). Photo (**C**) shows numerous alveolar macrophages that are immunohistochemically CD68 positive (100×). Photo (**D**) shows numerous desquamated pneumocytes in the alveoli that are immunohistochemically AE1AE3 positive (100×).

The autopsy findings confirmed all the clinically manifest symptoms of COVID-19 as the cause of death and also revealed damage to the vital organs, brain, heart, liver, and kidneys, as well as the lungs, as the primary targets of severe infection. Accordingly, the lungs of all the patients were edematous with acute or chronic pneumonia. For all the patients included in our study, the pathological changes in the lungs associated with COVID-19 caused interstitial pneumonia, and acute or chronic diffuse alveolar damage was revealed (Figure 1).

Superimposed bacterial sepsis caused by purulent pneumonia was found in three out of nine patients. Despite this, PMNs were found to be prominent in the blood vessels of every patient, but mainly in the lungs, brain, and heart. Given their pronounced presence in the blood, we assumed that they were located intravascularly due to endothelial damage caused by COVID-19 infection.

**Figure 2:**
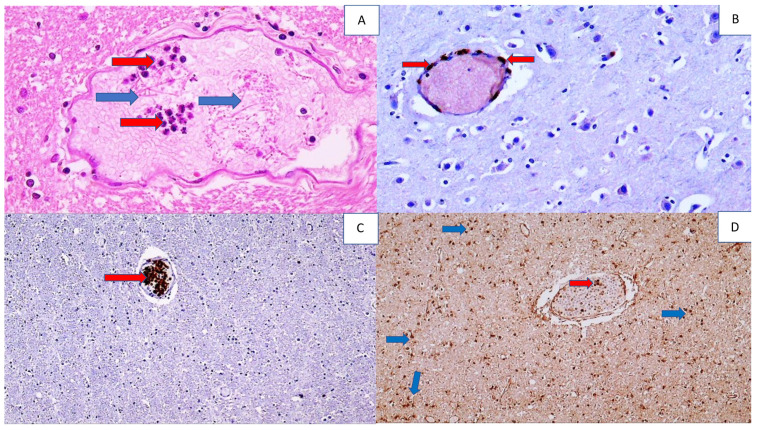
Histology and immunohistochemistry of a brain vessel with fibrin, PMNs, inflammatory T lymphocytes, and reactive astrocytes in the brain of a COVID-19 patient. Photo (**A**) shows a blood vessel in the brain filled with neutrophils (red arrows) and fibrin (blue arrows), (HE, 400×). Photo (**B**) shows a perivascular infiltrate of immunohistochemically CD3-positive T lymphocytes (arrow) in the cerebral vein (DAB, 200×). Photo (**C**) shows immunohistochemically MPO-positive PMNs in a cerebral blood vessel (arrow), (DAB, 100×), while Photo (**D**) shows immunohistochemically SOD2-positive diffuse reactive astrocytes in white matter of the brain (dark brown shows DAB staining; some are indicated by blue arrows) and inflammatory cells in a blood vessel (red arrow) (DAB, 100×).

In one patient with MIS, signs of encephalitis were found, where the edematous brain tissue contained predominantly perivascular CD3-positive T lymphocytes accompanied by PMNs within the blood vessels. In the majority of the remaining patients, pronounced cerebral edema with activation of reactive astrocytes, mostly perivascular, was found (Figure 2).

In a patient with disseminated intravascular coagulation (DIC), due to consumptive coagulopathy, perivascular bleeding with perivascular PMNs was observed. The finding of fibrin clots in the blood vessels of the brain was also associated with the intravascular finding of PMNs.

**Figure 3:**
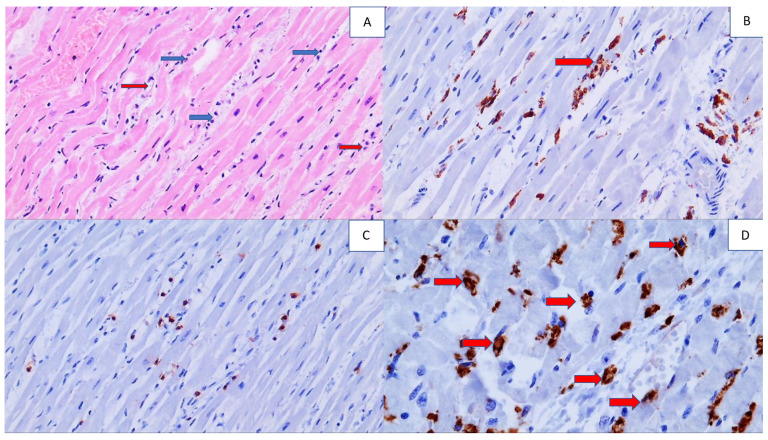
Histology and immunohistochemistry of the heart tissue of a COVID-19 patient with myocarditis. Photo (**A**) shows inflammatory cells (blue arrow) in edematous myocardial endomysium and the foci of myophagocytosis (red arrow) (HE, 200×). In Photo (**B**), immunohistochemically CD68-positive macrophages are present in the myocardium, being active in myophagocytosis (arrow), (DAB, 200×). Photo (**C**) shows immunohistochemically brown-stained CD3-positive T lymphocytes in the endomysium (DAB, 100×). Photo (**D**) shows an immunohistochemically positive reaction for MPO of PMNs in myocardial blood vessels and their spread in perivascular space (arrows), (DAB, 400×).

Myocarditis was found in two patients, in the child with MIS and in one elderly patient (Figure 3). The T lymphocytes were observed in a perivascular pattern and in edematous endomysia, while in the blood vessels they were accompanied by PMNs. In myocardial blood vessels, PMNs were present in the majority of the deceased patients, regardless of the superimposed bacterial infection.

**Figure 4:**
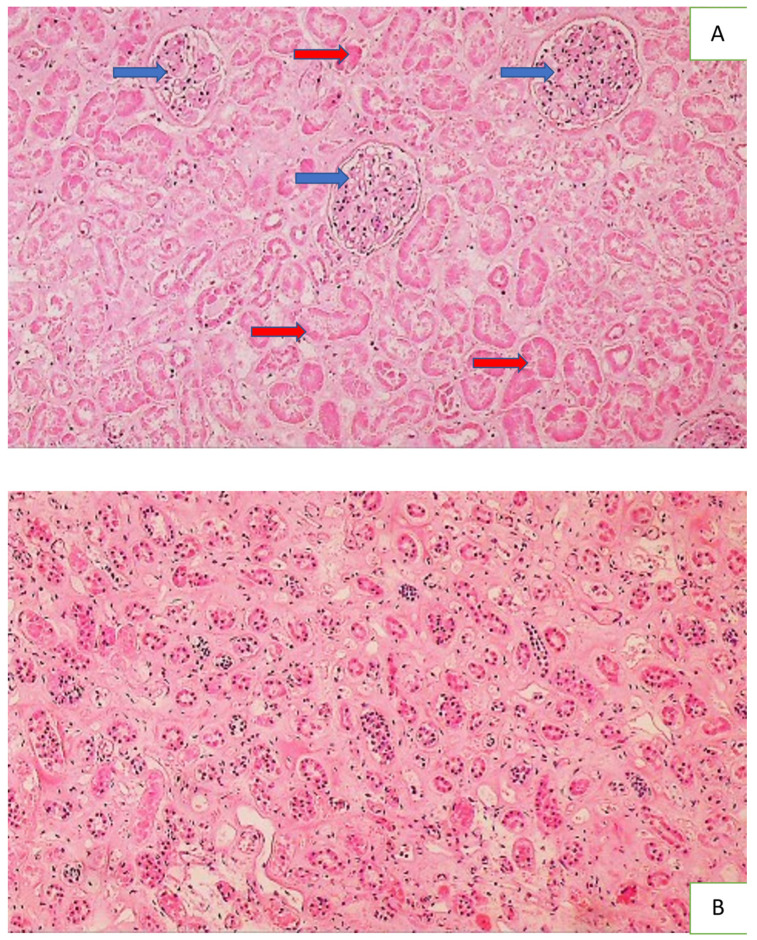
Histology of the kidney of a COVID-19 patient with acute tubular necrosis ATN. Photo (**A**) shows acute tubular necrosis in the renal tissue, presented by the necrotic epithelium (no cells with nuclei present) of the proximal and distal convoluted tubules (red arrows) and the preserved glomeruli (blue arrows) (HE, 100×), while Photo (**B**) shows the preserved epithelium containing the nuclei (dark spots) of collecting tubules (HE, 100×).

The majority of the deceased had renal acute tubular necrosis (ATN), with necrotization of the epithelium of the proximal and distal tubules, while structure of the glomeruli and collecting ducts was preserved. Apparently, the ATN occurred in most patients in the terminal stage of the disease as a component of multiple organ failure (MOF) and was not histologically manifest by tissue inflammation (Figure 4).

**Figure 5:**
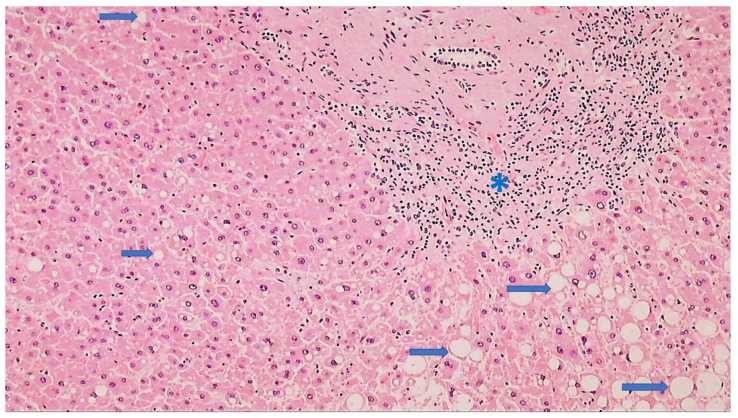
Histology of the liver of a COVID-19 patient with signs of steatosis (HE, 100×). Hepatocytes show lipid droplets of different sizes (arrows), while in the portal space, a moderate lymphocyte infiltrate (asterisk) is present.

Liver steatosis was found in the liver of the majority of the patients, while centrilobular cyanosis was found in two as a sign of cardiac insufficiency and dilatation of the right heart (Figure 5).

**Figure 6:**
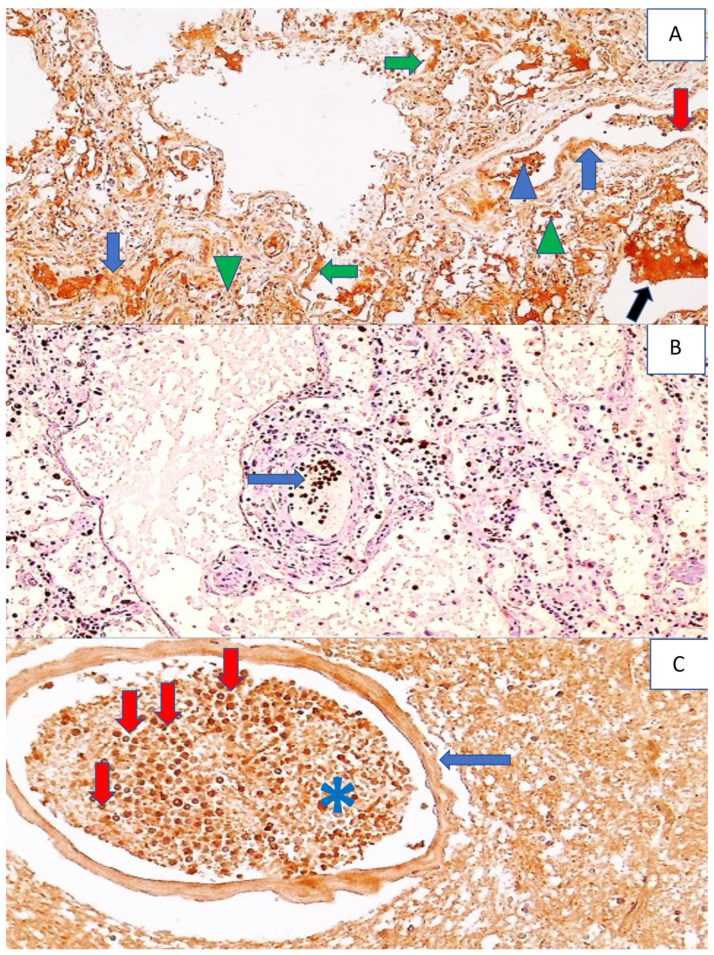
Immunohistochemistry for 4-HNE protein adducts and MPO in the lungs and 4-HNE protein adducts in the brain of a COVID-19 patient. Photo (**A**) shows the immunohistochemically positive reaction to 4-HNE in the pulmonary blood vessel walls (blue arrows) and in the content of the blood vessel (red arrow), in the edematous fluid in the alveoli (black arrow) and hyaline membranes (green arrows), in some inflammatory cells in the blood vessels (blue arrowheads), and in alveolar macrophages (green arrowheads), (DAB, 100×). Photo (**B**) shows an immunohistochemically positive reaction to MPO, exclusively in neutrophils that are mainly in the blood vessels of the lungs (arrow) and rarely outside them in the lung tissue, presented as dark brown spots, (DAB, 200×). Photo (**C**) shows a strong immunopositive reaction to 4-HNE in a blood vessel wall (arrow), in the content of blood vessels (asterisk), especially in the neutrophils within the blood vessel (red arrows), while being sparsely present in the surrounding edematous brain tissue, visible as dark brown cells (DAB, 400×).

**Figure 7:**
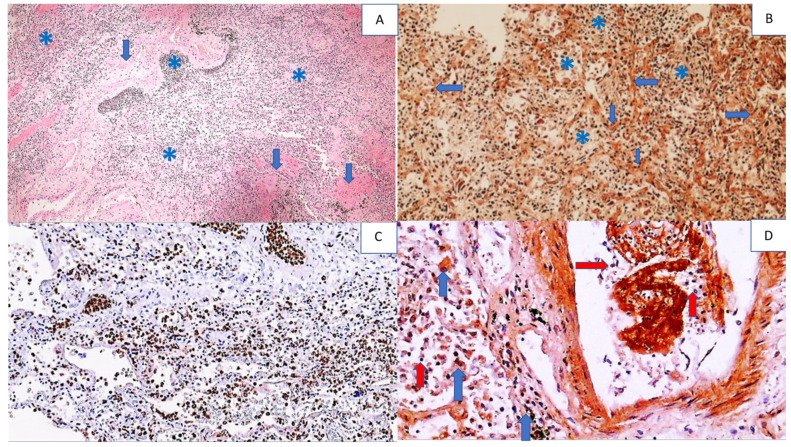
Histology and immunohistochemistry of the lungs of a COVID-19 patient with pneumonia and DAD. Photo (**A**) shows the destruction of pulmonary tissue with a barely recognizable structure due to severe inflammation (asterisks) and fibrosis (arrows), (HE, 50×). Photo (**B**) shows the immunohistochemical positivity for 4-HNE in the alveolar septa (arrows), edematous fluid, alveolar macrophages, and other inflammatory cells (asterisks) (DAB, 100×). In contrast, in Photo (**C**), immunohistochemical positivity for MPO is seen only for inflammatory cells stained brown (DAB, 100×). Photo (**D**) shows strong immunohistochemical 4-HNE positivity in some inflammatory cells, e.g., alveolar macrophages (blue arrows), while other inflammatory cells, such as mononuclear cells, in the vessel and lung tissue are negative 4-HNE-negative (red arrows) (DAB, 400×).

**Figure 8:**
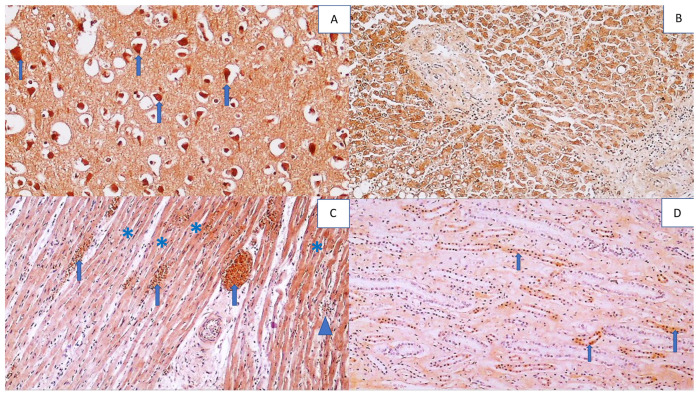
Immunohistochemical positivity for the 4-HNE protein adducts in the vital organs (except the lungs, presented by other figures) of the deceased COVID-19 patient. Photo (**A**) shows very strong immunohistochemical positivity for 4-HNE in neurons (arrows) within the edematous brain tissue (DAB, 400×). Photo (**B**) shows a pronounced intensity (but weaker than that in neurons) of 4-HNE positivity in liver cells (DAB, 200×). Photo (**C**) shows moderate to strong 4-HNE positivity in myocardial muscle fibers affected by myocarditis (asterisks), with strong 4-HNE positivity in myocardial vasculature (arrows) and some inflammatory cells (arrowhead), (DAB, 200×). Photo (**D**) shows the weakest intensity of 4-HNE immunopositivity in the epithelium of the renal tubules (arrows) (DAB, 200×).

A summary of the histological analysis and immunohistochemistry for 4-HNE protein adducts in the vital organs of patients deceased after aggressive COVID-19 are presented in Table 2 and Figure 6, Figure 7 and Figure 8.

4-HNE immunopositivity was observed in the endothelium, vascular wall, and vascular contents of all the organs affected by COVID-19 infection analyzed in this study, especially in the lungs and brain tissue (Figure 6).

Furthermore, immunohistochemical positivity for the 4-HNE protein adducts was observed in parenchymal cells: pneumocytes (Figure 7), hepatocytes, the epithelium of the renal collecting tubules, neurons, reactive astrocytes in the brain, and in cardiac muscle fibers and in the connective tissue of the lungs, liver, kidneys, and spleen and in reactive astroglia (Figure 8).

**Figure 9:**
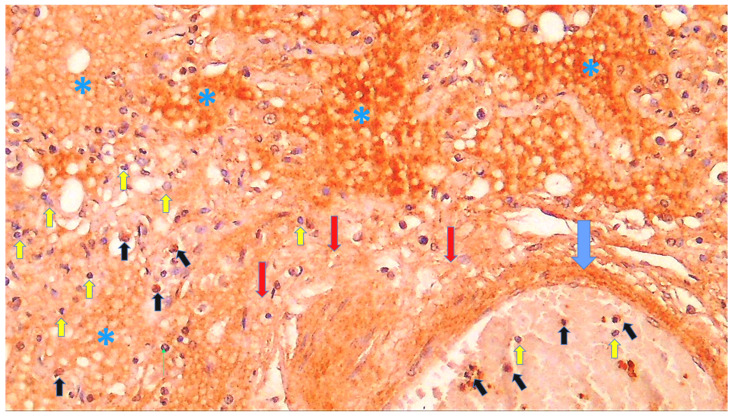
Immunohistochemistry for 4-HNE protein adducts in the lungs of a COVID-19 patient with pneumonia and DAD. There is a marked immunohistochemical positivity for 4-HNE present in the vessel wall (blue arrow), edematous fluid (asterisks), and interstitial stroma (red arrows). Some PMNs within the blood vessel and inflammatory cells in lung tissue are also positive (black arrows), while the majority are negative for 4-HNE, contrast-stained blue by hematoxylin (yellow arrows) (DAB, 600×).

In particular, 4-HNE was profound in the pulmonary blood vessels and edema, but only in some inflammatory cells (Figure 9).

Inflammatory cells, mainly PMNs, in the blood vessels or in the tissues of the analyzed vital organs were often 4-HNE immunopositive, as in the case of tissue macrophages, notably, alveolar macrophages. Lymphocytes, which were mostly CD3-positive T lymphocytes, were also, but much less often, 4-HNE- positive. The CD20-positive lymphocytes were found sporadically in the examined tissues, while CD138-positive plasma cells were mostly absent, although they were sporadically observed in the HE preparations. Due to only sporadic findings of B-lymphocytes and plasma cells, their HNE status could not be evaluated.

**Figure 10:**
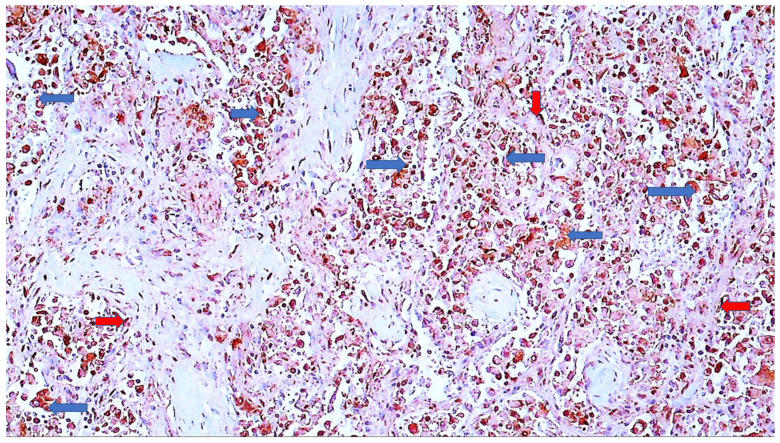
Immunohistochemistry for SOD2 of the lungs of a COVID-19 patient. Immunohistochemically positive reaction to SOD2 can be seen in alveolar macrophages (blue arrows) and pneumocytes (red arrows), while the rest of the tissue is negatively contrast-stained blue by hematoxylin (DAB, 600×).

SOD2 was occasionally immunohistochemically present in the endothelium and vascular content but was often observed in the smooth muscle cells of the blood vessel walls, especially in the lungs, where it was mostly associated with pneumocytes and alveolar macrophages (Figure 10).

**Figure 11:**
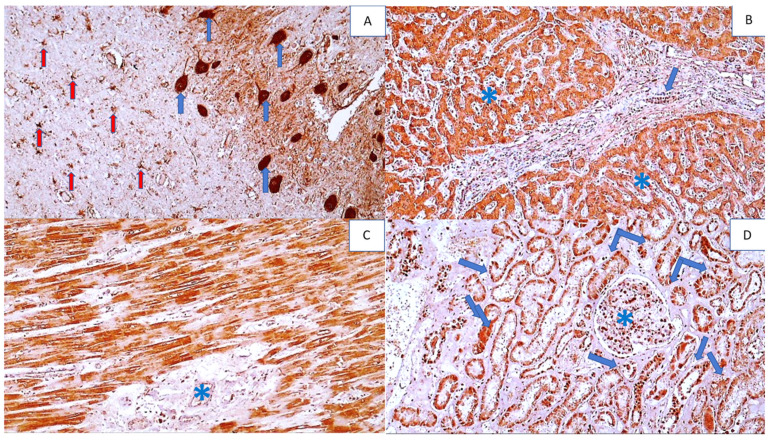
Immunohistochemistry for SOD2 adducts in the vital organs of a COVID-19 patient (except the lungs). Photo (**A**) shows strong immunohistochemical positivity for SOD2 in neurons (blue arrows) and reactive astrocytes (red arrows) in brain tissue (DAB, 400×). Photo (**B**) shows strong immunohistochemical SOD2 positivity in hepatocytes (asterisks) and bile duct epithelium (arrow) (DAB, 200×). Photo (**C**) shows strong SOD2 positivity in myocardial muscle fibers, but not in the endomysium (asterisk) (DAB, 200×), while Photo (**D**) shows SOD2 positivity in renal tubules (arrows) and glomerulus (asterisk) (DAB, 200×).

Furthermore, SOD2 was immunohistochemically revealed in the liver, both in hepatocytes and the epithelium of the bile ducts, heart muscle fibers, neurons, and reactive astrocytes within the brain, and in glomerular cells and the renal collecting ducts, strongly resembling 4-HNE-immunopositivity (Figure 11).

However, while SOD2 immunopositivity was strongly pronounced in the kidney, including the glomeruli and all types of tubules, and was positive in the majority of alveolar macrophages in interstitial pneumonia and DAD, it was very rarely detected in PMNs, inflammatory lymphocytes, and blood vessels, being absent in edematous tissues, the extracellular matrix, and connective tissue.

## 4. Discussion

The results of this study summarize the most important common autopsy findings of patients who died after aggressive COVID-19 and confirm our preliminary findings on 4-HNE as an important factor of SAR-CoV-2 infection causing sepsis-like systemic vascular and oxidative stress [16]. Namely, 4-HNE protein adducts were found in all the vital organs and were associated especially with inflammation, edema, and tissue destruction. This was most prominent in the lungs and brain, while in the other vital organs analyzed, 4-HNE was less pronounced. While 4-HNE was almost always present in the blood vessels and the vessels’ wall, it was not always detected in the inflammatory cells. These findings suggest that origin of 4-HNE could be blood rather than the oxidative burst of inflammatory cells, thus supporting several recent papers.

The radiologic–pathologic correlation of the lungs of 14 patients who died from lethal COVID-19 showed diffuse alveolar damage and capillary congestion together with microthrombosis of the lungs, suggesting that vascular alterations could be a key pathogenic factor in lethal COVID-19 [32]. Similar conclusions were also given by several papers that revealed vascular stress as a crucial pathologic principle of lethal COVID-19 [33,34,35,36,37]. The lipid peroxidation product 4-HNE might indeed be among the triggers of such systemic stress, especially in the case of elderly people who developed atherosclerosis, which is associated with the accumulation of the likely reversible 4-HNE protein adducts [38]. 4-HNE is not only associated with atherosclerosis but also with obesity, altering the lipid metabolism and accumulating in adipose tissue; although 4-HNE is not locally generated by the adipocytes, it penetrates adipose tissue from the blood, eventually being regulated by insulin and metformin [39]. It should also be mentioned that 4-HNE can cause damage to the endothelium [40]; a similar finding was observed in the vessels of COVID-19 patients, while the accumulation of 4-HNE in the aorta is the most prominent from 60–65 years of age [38], which was exactly the age range of the majority of the patients in our study, whose blood vessels of all the vital organs were loaded with 4-HNE. However, since similar findings in our study were also observed for the eleven-year-old child, it is not certain how crucial the accumulation of 4-HNE in the vessels is for the lethal outcome of aggressive SARS-CoV-2 infection.

On the other hand, the relevance of lipid metabolism for the pathogenesis of COVID-19 is stressed by many researchers [41,42,43]. The recently revealed downregulation of the low-density lipoprotein (LDL) metabolism in peripheral blood mononuclear cells of patients who survived severe COVID-19 [42] might be especially important, thus complementing our preliminary findings that have shown a correlation between the level of autoantibodies again oxidized LDL and 4-HNE protein adducts in the blood of COVID-19 patients who did not survive the disease [16]. This is not surprising because 4-HNE-modified epitopes dominate in the oxidized LDL [44]. Accordingly, we assume that lipid peroxidation affects LDL as a substrate, consequently generating 4-HNE that can be further transported in the form of albumin adducts [45]. This option could explain why a similar pattern of 4-HNE-immunohistochemsitry was found even in the absence of age-related atherosclerosis on the one hand, while on the other hand, this could also offer an explanation for the sensitivity of the obese patients to severe SARS-CoV-2 infection because the levels of the 4-HNE protein adducts in the blood are increased in obese people, even in the absence of any clinically manifest disease [45].

It is likely that the crucial stage of severe COVID-19 is self-catalyzed lipid peroxidation, which is manifested by systemic vascular and oxidative stress [46,47] and may trigger even more inflammation, forcing the innate immune system to increase a further release of cytokines and destructive enzymes manifested as sepsis [48,49,50,51]. Therefore, the lipid peroxidation trigger and 4-HNE mediated systemic vascular and oxidative stress could be associated with an uncontrolled (auto)immune stress response, which may lead to ARDS and MOF, as observed in our patients. The high importance of the malfunctioning immune system destroying vital organs, not only the lungs but even the brain, for severe/lethal SARC-CoV-2 infection is well known, even if the virus itself may not always be the cause [52,53,54], as is also the importance of uncontrolled oxidative stress [55,56]. Both of the supporting findings of our study suggest that lipid peroxidation, notably, 4-HNE, might be the link between these two vicious circles of lethal COVID-19.

Our study revealed that the SOD2 might be another very important link between a malfunctioning immune system and oxidative stress, alongside 4-HNE. Namely, the immunohistochemical resemblance of the findings observed for 4-HNE and SOD2 in all the vital organs of our patients indicated a possible pathogenic interference between this crucial antioxidant enzyme specific for mitochondria (SOD2) and 4-HNE regulating mitochondrial RSO production, stability, and leakage [52]. Consequently, this could result in Fenton’s reaction, ferroptosis, and the spread of lipid peroxidation, shown by the abundant immunostaining for both biomarkers.

However, immunostaining for SOD2 was limited to the cells, while 4-HNE was found diffusely present, although not in every inflammatory cell. Furthermore, SOD2 immunopositivity was strongly pronounced in all types of kidney tubules but was very rarely detected in PMNs, inflammatory lymphocytes, and blood vessels, being absent in edema, the extracellular matrix, and connective tissue. These findings demonstrate major differences in 4-HNE’s appearance, which was especially pronounced in the blood vessels and extracellular fluid (edema); it was always present in connective tissue and was usually present in the inflammatory cells (although not always), but was only weakly present in the kidneys, being limited to the epithelium of the collecting tubules.

Further detailed searches for the differential expression of 4-HNE and SOD2 might be a target for future research aiming to better understand the interference between lipid peroxidation and inflammatory cells generating vascular and systemic oxidative stress in lethal COVID-19.

## Figures and Tables

**Table 1:** Major clinical and autopsy findings of patients deceased after lethal COVID-19.

Age	Sex	Comorbidity	Diagnoses	Organ	Tissue Findings	Inflammatory Cells *	Bleeding	Thrombosis	Edema
58	M	DM2 ^1^	ARDS ^2^	lungs	DAD ^3^, interstitial pneumonia	Ly, Pl, Ma, PMN ^4^	no	yes	yes
		Hypertension	pulmonary emboly pneumonia interstitialis	heart	myocardial fibrosis atherosclerosis	PMN	no	no	yes
		Steatosis hepatis	atherosclerosis						
			dilatatio cordis	brain	brain edema	PMN, Mo	yes	no	yes
54	M	Obesity Hypertension	ARDS (DAD)	lungs	DAD, inerstitial pneumonia, DIC	Mo, PMN	yes	no	yes
			pneumonia						
			DIC ^5^	brain	edema, DIC	PMN, Mo	yes	yes	yes
			myocardial fibrosis hypertrophia and dilatatio cordis	heart	myocardial fibrosis	Mo	no	no	no
			steatosis hepatis renal ATN ^7^	liver	steatosis hepatis	Mo	no	no	no
55	M	Hypertension	(sepsis staphylococcica) ARDS (DAD)	lungs	DAD, pneumonia abscedens	PMN	no	no	yes
			pneumonia abscedens	brain	brain edema	Ly. PMN, Mo	no	no	yes
			steatosis hepatis	liver	steatosis hepatis	Ly, PMN	no	no	no
			atherosclerosis heart dilatation	heart	myocardial fibrosis	PMN	no	no	no
57 ^6^	M		(sepsis *A. baumanii*), ARDS (DAD)	lungs	pneumonia chronica and suppurativa, DAD	Ma, Mo, Ly, Pl, PMN	yes	yes	yes
			pneumonia	kidney	renal ATN	PMN	no	no	no
			renal ATN	heart	myocardial fibrosis and lipomatosis	Ly	no	no	no
			myocardial fibrosis	liver	centrilobular cyanosis	Ly	no	no	no
				brain	brain edema	Ly, Mo	no	no	no
65	M	DM2	bilateral pneumonia	heart	myocardial fibrosis	Ly, Mo, PMN	no	no	no
		Hypertension Hyperlipidemia	pulmonary microthrombosis	lungs	lung fibrosis, Chronic DAD, acute hemorrhage	Ma, PMN	yes	yes	yes
		Atherosclerosis	atherosclerosis	kidney	renal ATN	PMN	no	no	no
		IBD ^8^	heart hypertrophia	liver	steatosis hepatis	Ly, PMN	no	no	no
			myocardial fibrosis	heart	myocardial fibrosis	PMN	no	no	no
				brain	brain edema	Ly	no	no	no
11	M		MIS ^9^, ARDS (DAD)	lungs	ARDS (DAD)	Mo, Ly	yes	no	yes
			myocarditis encephalitis	heart	myocarditis				
			edema cerebri	brain	encephalitis, edema	PMN, Ly, Ma	no	no	no
			MOF ^10^, renal ATN	kidney	renal ATN	PMN	no	no	no
			centrilobular liver necrosis	liver	autolysis	PMN, Mo	no	no	yes
74	F	DM2	ARDS						
		Hypertension	steatosis hepatis myocardial fibrosis	liver	hepatic centrilobular necrosis	Ly	no	no	no
			heart dilatation						
			pneumonia	lungs	DAD, interstitial pneumonia	PMN, Ma	yes	no	yes
			renal ATN	kidney	renal ATN	PMN	yes	no	yes
			myocardial fibrosis	heart	myocarditis	PMN, Ly	no	no	no
65 ^6^	M	Atherosclerosis	ARDS (DAD) pneumonia	lungs	DAD, pneumonia suppurativa,		no	yes	yes
		Hypertension	hematoma intracerebrale	brain	acute bleeding and brain edema	PMN, Ly	yes	no	yes
		Obesity	hematocephalus						
			myocardial fibrosis	heart	myocardial fibrosis	PMN	no	no	no
75	M	Atherosclerosis	ARDS (DAD)						
			interstitial pneumonia	lungs	pneumonia, DAD	PMN, Mo, Ly	no	no	yes
			myocardial fibrosis, heart dilatation and hypertrophy	heart	myocardial fibrosis	PMN, Ly	no	no	no
			renal ATN	kidney	renal ATN	PMN	no	no	no

^1^ diabetes mellitus type 2; ^2^ acute respiratory distress syndrome; ^3^ diffuse alveolar damage; ^4^ lymphocytes (Ly), plasma cells (Pl), macrophages (Ma), polymorphonuclear leukocytes (PMN), monocytes (Mo); ^5^ disseminated intravascular coagulation; ^6^ patient was supported by extracorporeal membrane oxygenation (ECMO); ^7^ acute tubular necrosis; ^8^ inflammatory bowel disease; ^9^ multisystem inflammatory syndrome; ^10^ multiple organ failure; * inflammatory cells that were the most pronounced.

**Table 2:** Summary of the histological analysis and immunohistochemistry for 4-HNE protein adducts in the vital organs of patients deceased after aggressive COVID-19. The most common findings observed for all patients or for patients whose respective vital organs were most severely affected by SARS-CoV-2 infection are summarized.

	Organ Structure/Tissue Appearance	Inflammatory Cells	Immunohistochemistry for 4-HNE Protein Adducts	SOD2 Immunohistochemistry
Lungs	Edematous Diffuse alveolar damage Acute and chronic interstitial pneumonia	All types of leukocytes—mostly PMNs within blood vessels, otherwise mostly pulmonary macrophages and T lymphocytes	Very strong positivity of the blood vessels, pneumocytes, hyaline membranes, and edematous liquid Some inflammatory cells positive, mostly PMNs, alveolar macrophages positive	Observed in the smooth muscle cells of the blood vessel walls and less common in the endothelium and vascular contents Prominent in alveolar macrophages in interstitial pneumonia and DAD PMNs negative
Heart	Often fibrotic Occasional myocarditis	T lymphocytes around blood vessels and in edematous endomysia PMNs in the blood vessels	Strong positivity of blood vessels Positive cardiac muscle fibers Positive inflammatory cells	Positive muscle fibers
Brain	Edematous	Perivascular CD3-positive T cells PMNs and T lymphocytes within the blood vessels	Strong positivity of blood vessels and vascular content Very strong and diffuse immunopositivity in neurons Positive astroglia	Positive neurons and reactive astrocytes
Liver	Often steatosis Rarely centrilobular cyanosis	Not prominent	Positivity of the blood vessels Positive hepatocytes	Positive hepatocytes and epithelium of the bile ducts
Kidneys	Acute tubular necrosis, with necrotization of the epithelium of the proximal and distal canals Structure of the glomeruli and collecting ducts preserved	Inflammatory cells were not found	Strong positivity, but only in collective tubules	Positive glomerular cells and collecting ducts

## Data Availability

Not applicable.

## Supplementary Materials

The following supporting information can be downloaded at: https://www.mdpi.com/article/10.3390/cells11030444/s1, Figure S1: Supplementary Figure with negative control immunohistochemistry slides, processed without primary antibodies.

## Abbreviations

COVID-19—Coronavirus disease 2019; SARS-CoV-2—Severe acute respiratory syndrome coronavirus; SARS—Severe acute respiratory syndrome; 4-HNE—4-Hydroxynonenal; ROS—Reactive oxygen species; NLRP3—Nucleotide-binding oligomerization domain-like receptor P3; ERK—Extracellular signal-regulated kinases; SOD2—Superoxide dismutasetype 2; MPO—Myeloperoxidase; HO-1—Heme oxygenase; ECMO—Extracorporeal membrane oxygenation; PBS—Phosphate buffered saline; DAB—3,3,-diaminobenzidine; HE – hematoxylin eosin staining; MIS—Multiple inflammatory syndrome; MOF—Multiple organ failure; DM2—Diabetes mellitus type 2; DAD—Diffuse alveolar damage; Ly—Lymphocytes; Pl—Plasma cells; Ma—Macrophages; PMN—Polymorphonuclear leukocytes; Mo—Monocytes; DIC—disseminated intravascular coagulation; ATN—acute tubular necrosis; IBD—inflammatory bowel disease; LDL—low-density lipoprotein.
