# Supplementary material for: Post-mortem Findings of Inflammatory Cells and the Association of 4-Hydroxynonenal with Systemic Vascular and Oxidative Stress in Lethal COVID-19
Source: Cells. 2022 Jan 27;11(3):444. doi: 10.3390/cells11030444 (PMC8834180; doi:10.3390/cells11030444)
Supplement: Supplementary file 1 [file cells-11-00444-s001.zip › cells-1557895-supplementary.pdf]

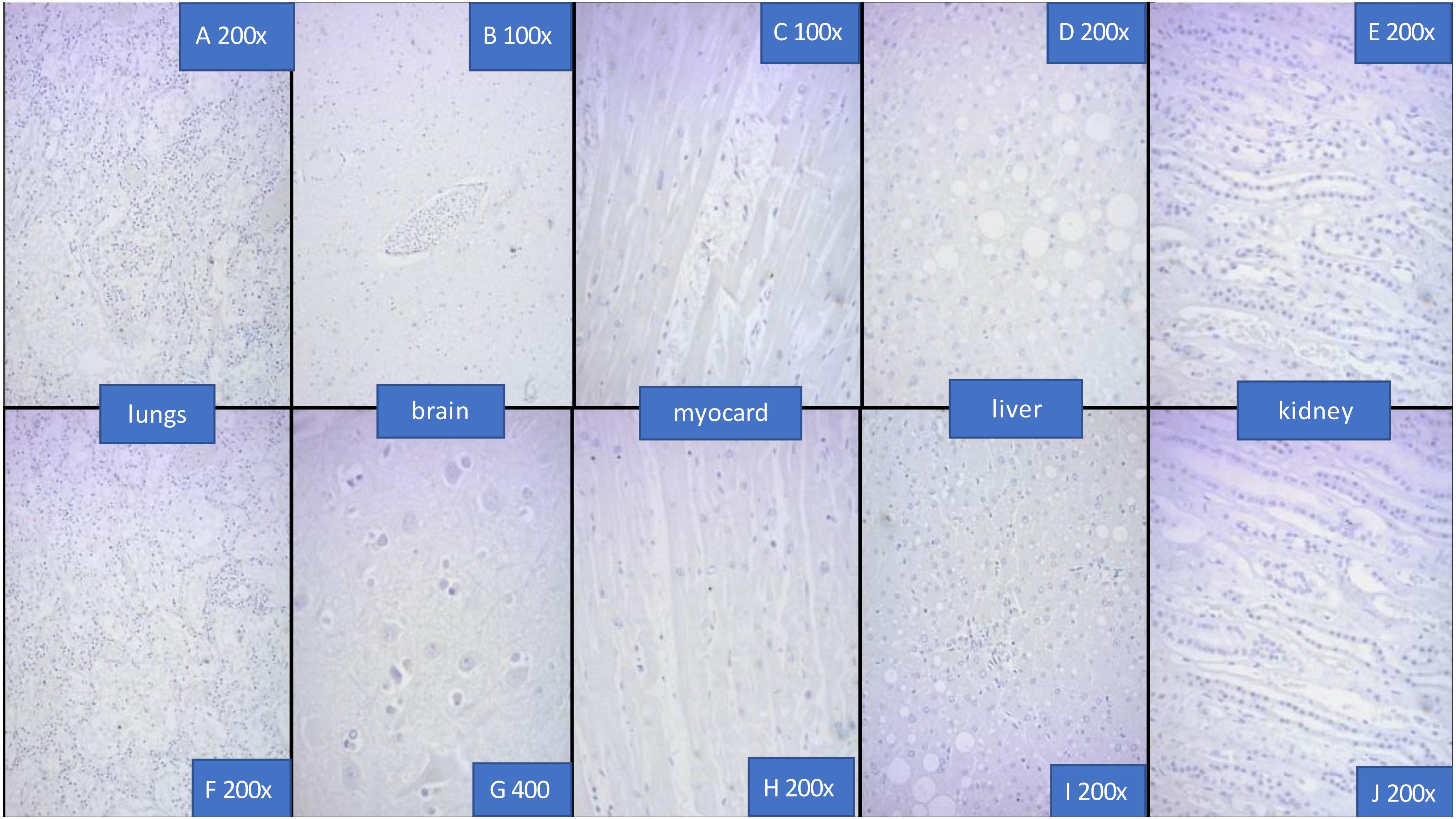


Figure S1. Supplementary Figure with negative control immunohistochemistry slides, processed without primary antibodies. Photos A, B, C, D, and E show negative controls that were obtained while immunohistochemistry was done for 4-HNE. Photos F, G, H, I, and J show negative controls that were obtained while immunohistochemistry was done for SOD2. The organs analysed and the magnifications used are indicated on the Figure.
